# Protein stability governed by its structural plasticity is inferred by physicochemical factors and salt bridges

**DOI:** 10.1038/s41598-020-58825-7

**Published:** 2020-02-04

**Authors:** Anindya S. Panja, Smarajit Maiti, Bidyut Bandyopadhyay

**Affiliations:** 1 Post Graduate Department of Biotechnology, Molecular informatics Laboratory, Oriental Institute of Science and Technology, Vidyasagar University, Midnapore, West Bengal India; 2 Post Graduate Department of Biochemistry and Biotechnology, Cell and Molecular Therapeutics Laboratory, Oriental Institute of Science and Technology, Vidyasagar University, Midnapore, West Bengal India

**Keywords:** Molecular evolution, Protein folding

## Abstract

Several organisms, specifically microorganisms survive in a wide range of harsh environments including extreme temperature, pH, and salt concentration. We analyzed systematically a large number of protein sequences with their structures to understand their stability and to discriminate extremophilic proteins from their non-extremophilic orthologs. Our results highlighted that the strategy for the packing of the protein core was influenced by the environmental stresses through substitutive structural events through better ionic interaction. Statistical analysis showed that a significant difference in number and composition of amino acid exist among them. The negative correlation of pairwise sequence alignments and structural alignments indicated that most of the extremophile and non-extremophile proteins didn’t contain any association for maintaining their functional stability. A significant numbers of salt bridges were noticed on the surface of the extremostable proteins. The Ramachandran plot data represented more occurrences of amino acids being present in helix and sheet regions of extremostable proteins. We also found that a significant number of small nonpolar amino acids and moderate number of charged amino acids like Arginine and Aspartic acid represented more nonplanar Omega angles in their peptide bond. Thus, extreme conditions may predispose amino acid composition including geometric variability for molecular adaptation of extremostable proteins against atmospheric variations and associated changes under natural selection pressure. The variation of amino acid composition and structural diversifications in proteins play a major role in evolutionary adaptation to mitigate climate change.

## Introduction

Modifications in protein structures from organisms that have evolved under extreme environmental conditions differ in how they maintain optimum activity. For example, in the case of halophiles, their optimal growth is associated with their optimal metabolic functions. Heat tolerant organisms are classified as thermophiles, which have optimum growth temperature (OGT) in the range of 45 °C–80 °C and hyper thermophiles with OGT of above 80 °C. Psychrophiles are the organisms which grow on cold condition, that have OGT below 10 °C. Alkalophiles are found in an alkaline p^H^ of more than 9. Alkalophiles and haloalkaliphiles are isolated from extremely alkaline-saline environments, alkaline soil and film such as the Western soda lakes of the United States and Rift valley lakes from East Africa, these are also available from natural environments^1,2^. Most of the acidophilic microorganisms survive in low pH by modifying their intracellular protein along with their genome. Evolutionarily conserved protein structures and their sequences showed similarities in their functions but often they differ in their sequence pattern^3^. Crystallographic and NMR structures sometimes differ from each other due to their specific experimental condition and retrieval oucome^4–7^. The report revealed that proteins with >40% sequence identity may also represent structural homology amongst themselves^8^.

Organisms that survive at very high temperatures were systematically studied after the discovery of *Thermus aquaticus* from a hot spring of yellow stone^9^. The physiochemical adaptive mechanisms have indicated that high hydrophobic core^10,11^, closest loops^12^ and compactness occur due to the presence of the small amino-acid residues^13^, which are probably the main factor for stress adaptability. The presence of higher frequencies of polar amino acids at the surface and non-polar amino acids in the core region^14^ results in increased hydrogen bonding, isoelectric points^15^ and salt bridges, which may in turn confer greater thermostability^16^. In our previous work, it was reported that Gly, Val, Ala are generally preferred in thermophiles^17^, whereas, Gln, His, Met, Cys are preferred in mesophilic proteins. Subtle differences are observed between the sequences and structures of thermophilic and mesophilic proteins, despite the fact that their orthologs share same catalytic mechanisms^18^. In some cases, structures are similar but corresponding sequence of thermostable and mesostable proteins are significantly different^19^. Due to the hydrophobicity effect, psychrophilic proteins are more stable during cold denaturation process^20^. Psychrophilic protein with catalytic multi domains are reported to be heat liable in nature^21^. Lesser salt bridges in the outer surface of a protein results in reduced conformational flexibility^22,23^. Halo-tolerant organisms can survive either in high salinity or in low salinity^24^. Apart from these, most of the acidic residues with salt bridges on the surface of the protein make peripheral region more hydrophilic and flexible. The hydrophobic pockets of the halophilic proteins are less exposed to specific molar concentration of inorganic salts but these are shown to be more propinquous to organic salts^25^. Evolutionarily, all these modifications in the protein sequences help to study the mechanisms by which organisms adapt to high-salinity conditions. The stability of protein in alkaline condition is maintained by decreasing the number of negatively charged amino acids (Asp, Glu) and increasing the number of neutral hydrophilic amino acids (His, Asn, Gln, and Arg)^26–29^. The simultaneous participation of positively charged and negatively charged residues in salt bridges contributes to the stability of proteins in alkaline environment^30^. Acidophiles utilize sulfide minerals into their metabolic processes. Acidophiles are able to grow not only in low pH but also in metal rich condition^31–33^. In 2004, Schafer *et al*. concluded that, although an enrichment of proline residues plays a significant role in the protein thermo-stability, this is not the issue in case for proteins in acidic environments^34^. Excess of Glu and Asp on the outer surface of some enzymes and proteins can enable these proteins to perform optimally in low pH environment. Lower isoelectric point and the minimum negative charge could help to stabilize the bond in acidic environment^35^. During the verification attempt at ultrahigh resolution, protein structures were precisely analyzed and certain level of peptide nonplanarity was observed by Rosetta in 2013, but shorter peptide do not show no significant nonplanarity^36,37^. We also observed that the non-planarity is one of the major determinants in protein stability which is increasingly abundant in protein carboxy-terminal at increasing temperature^38^.

Here, we designed an extensive series of *in silico* studies to thoroughly survey high-resolution protein structures and their sequences in order to explore the impacts of extreme environments on the protein stability. The percentage of total residues, pair wise sequence alignment score (PSA), structural differences by root mean square deviation (RMSD) and other physicochemical parameters of extremostable and non-extremostable proteins were analyzed in the present study^39–41^

## Result

### Physiochemical property along with amino acid composition of halophilic, acidophilic, alkalophilic, thermophilic, psychrophilic and their corresponding homologous normal protein

**Figure 1:**
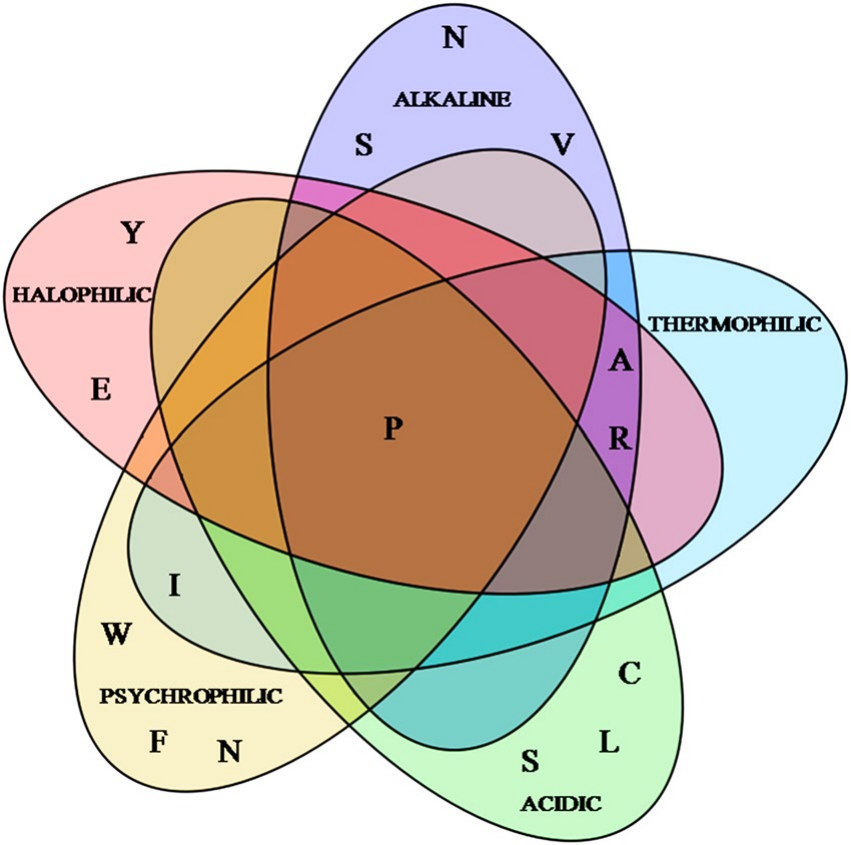
Representation of principal amino acid from the global composition of of acidic, alkaline, halophilic, thermophilic and psychrophilic proteins.

We analyzed the relative abundance of twenty amino acids in twenty-two alkaline and non alkaline, fourteen acidic and non acidic, thirty halophilic and non-halophilic, twenty-three thermophilic, eight psychrophilic and their homologous mesophilic proteins. In our present study, a list of all the proteins is provided in the Supplementary Material (Supplimentary 1 Table A-J). The fraction of amino acids distribution plot from various categories is shown in Fig. S1. The X axis represents the category of amino acids residual preference that is 0–2%, 2–4% and so on. The Y axis represents the total number of sample and compared with an average amino acid residue in their polypeptide chain. It was observed that more than 80% of halophilic, thermophilic, alkalophilic, acidic proteins showed a trend of having a higher amount of glycine, alanine, and isoleucine. Alkaline, psychrophilic and halophilic proteins showed higher amount of isoleucine in there sequences. The result from Fig. S1 represented a trend of having a large amount of neutral non polar aliphatic amino acids like glycine, alanine, valine, isoleucine in all the proteins studied. Around 25% acidic proteins, 22% thermophilic proteins and 18% alkaline with psychrophilic proteins contained more proline (8–10%) in their polypeptide chain (Fig. S1). The hydrophobic neutral non polar amino acid valine was abundant (25%) in alkaline and halophilic proteins in their chain with >12% of abundance. Major polar amino acids glutamate, threonine, cysteine were exhibited significant higher level in most of the non-acidic, non-alkaline, non-halophilic and mesophilic proteins. Most of the neutral proteins constituted a higher occurrence rate (0–4%) of cysteine, threonine (>10%) in non-alkaline, non-halophilic and non-acidic conditions. In the case of psychrophilic proteins, a higher number of neutral hydrophilic aspargine and serine were there and a little higher hydrophobic, non-polar, aromatic tryptophan and phenylalanine were also observed. The statistical significance of these occurrence rates of amino acids contributed a higher amount of hydrophilic charged Glutamate in halophilic and alkaline proteins. Relatively opposite mesophilic and non-acidic proteins (25%) showed some higher percentage of glutamic acid and that is displayed in Fig. 1.

### Structural stability based on polar, non- polar amino acids, isoelectric point and expression hydrophobicity

The periodicities of polar and non polar residues were conferred structurally and functionally stable due to the avoidance of water (Fig. S2). Non-polar amino acids burial into their hydrophobic pockets was noticed above 55% in acidic, 60% in alkaline and thermostable protein samples. Some higher amounts (Fig. S2) of polarity were shown by halophilic proteins to attract aqueous solvent for better interaction in their folding. These properties may be a potential parameter to estimate protein’s functional and structural stability^42^. An increase in the proportion of hydrophobicity in the protein has indicated the presence of higher number of the smallest non polar amino acids like alanine, glycine and lyophilic valine, which was positively correlated with our result. Our observations indicated that in stressful conditions hydrophobicity is promoted in extremostable proteins with respect to their homologoue counterpart. Statistical analysis of the Students t-test at 5% significant level indicated that a less significant difference exists between extremophile and non-extremophile proteins in some of the physicochemical parameters Whereas, higher significant difference found in isoelectric point in both acidic and halophilic proteins were found p < 0.009^14,16^. Only in case of halophilic proteins charge showed higher deviation p < 0.005. Charge distribution on the protein surface has been linked to higher degree of polar solvent-association by polar amino acids. This increases the protein adaption by increasing the interactibility of protein to its substrates/ligands^17^.

In the current study we found more hydrophobicity (45–55%) in halophilic, 45–59% in acidic and 49–53% in alkaline with thermophilic proteins respectively^11^ whereas, it was little higher in psychrophilic proteins. Due to the presence of charged amino acids, the isoelectric point (Fig. S2) showed a significantly lower value in extremostable proteins compared to non-extremostable counterpart P < 0.0003 and P < 0.01 respectively. Increased proportion of hydrophobic region in the proteins makes it well folded and generates more grooves to avoid water molecule. Furthermore, the contrasting pattern of hydrophobic and hydrophilic regions make the balance of protein surface association as well as interior core formation^11^. This balance increases the interaction ability of the protein to its surrounding medium; at the same time it increases the ligand binding capacity in the core region. Phenotypically, a better adapted organism basically carries a greater proportion of adaptable proteins^11,25^. A large interior core enables a protein to generate more ligand binding site, and hence more interaction. Increased extent of cross-talk helps the proteins link with the different metabolic processes for better and optimized stability in extreme environments.

### Sequential and structural diversity

**Figure 2:**
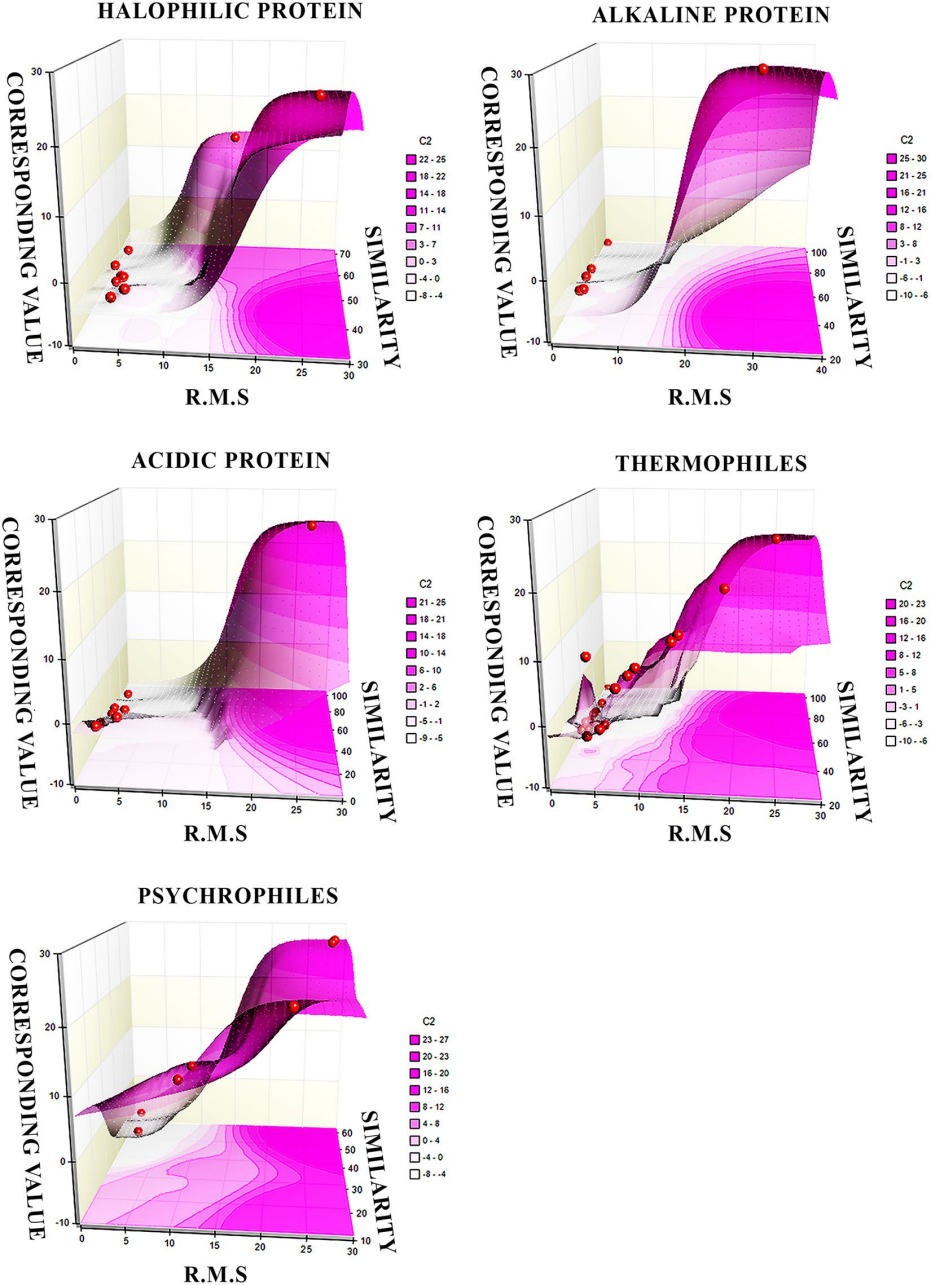
Plots of 3D response surface, displaying correlation of sequence similarity (PSA global) and structural similarity (RMSD) values between extremostable and non-extremostable proteins.

We performed pair wise (PSA) global alignment of the protein sequences with the help of emboss needle in ebi website (http://www.ebi.ac.uk/Tools/psa) and their corresponding structure were analyzed (RMSD) in pymol software. When we compared pairwise sequence alignment (PSA) values with structural alignment RMSD values, the negative correlation value indicated that in contrast to sequence structure relation of both extemostable and non-extremostable proteins are insignificant (Table 1). Halophilic (1MOG) and non-halophilic (2CZ8) proteins dodecin showed 0.69 RMSD value but sequence identity showed 36.8% only. Nucleoside diphosphate kinase of both halophilic (2AZ1) and non-halophilic (1EHW) proteins showed high RMS deviation 15.86 but sequence alignment showed little higher score 50.3%. Similarly, another acidic protein (1BAS) Fibroblast growth factor-2 showed 0.656 RMSD value with non-acidic (1AXM) proteins but showed only 47% sequence based identity. Interestingly, an acidic (1E9Y) and non-acidic (1IE7) proteins urease subunit beta protein showed 0.856 RMSD value but 24.4% in case of sequence identities, represented in Supplementary File (U-Y in S3). Likewise, most of the thermophilic, mesophilic, psychrophilic, alkaline and non alkaline proteins showed an unusual relation between structural and sequential similarity (Fig. 2).

**Table 1:** Discriminatory power represented by correlative distribution derived from sequence identity with structural RMSD of the extremophiles and non-extremophiles protein structure of Acidic(A) vs Non-acidic(NA), Alkaline(ALK) vs Non-alkaline(NALK), Halophilic(H) vs Non-halophilic(NH), Thermophiles(T)/Psychrophiles(P) vs Mesophiles(M).

	A vs NA	ALK vs NALK	H vs NH	T vs M	P vs M
Correlation value	+0.282549302	−0.413923377	−0.0755	−0.326803218	+0.057274913

### Salt bridge formation for stability

Certain acidic residues play a major role to stabilize proteins in stress tolerant organisms, which is protected by solute ions through structural rigidity. Dielectric properties of protein surface stabilize protein structure. We observed slightly higher number of salt-bridge interactions in stress-exposed proteins than their normal counterparts (Fig. S4). The slight increase is consistent with the probabilistic advantage of salt bridges in greater stress-exposed proteins^17,43^.

### Structural exploration

**Figure 3:**
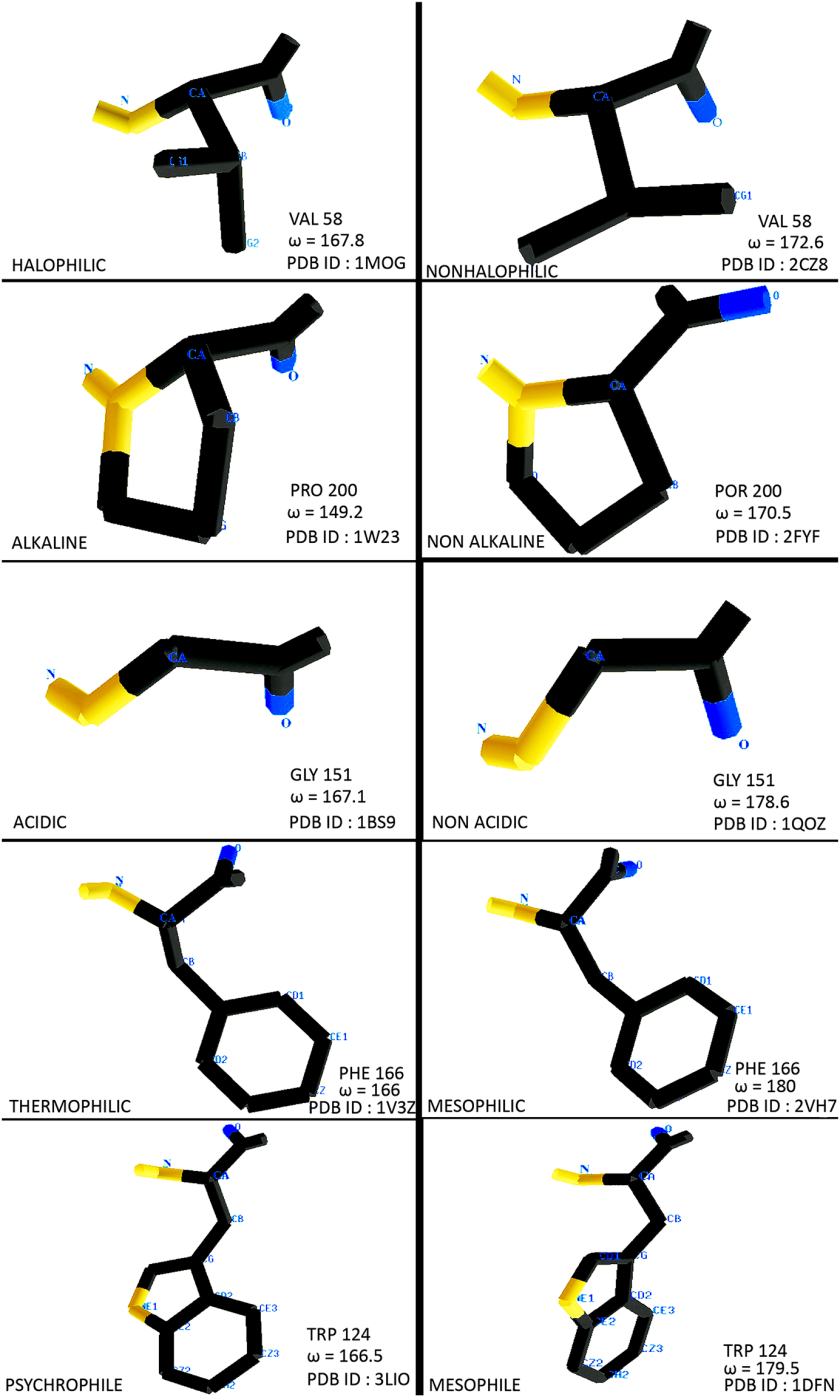
Schematic representation of planar and non planar amino acids describing ω angle.

The structures of all the proteins were investigated on the basis of their experimental resolutions. Using Vadar tools, Ramachandran plot was assessed (Table S1)^44^ and explored all the proteins by investigating the dependence of phi/psi/omega angles on the peptide conformation. Residues of the stress tolerant proteins were more condensed in the core area of beta sheet, right-handed and left-handed helix locations (Fig. S5). High number of non-planarity was represented by positive charge Arg, negative charge Asp and by few neutral amino acids like Val, Leu, Gly, and Phe. The conformational properties of both sides (ω_a_ for above and ω_b_ for after) amino acids correspond to non polar amino acid was extensively investigated. Non polar amino acids were preferred in the vicinity of non-planer amino acids. But exceptionally higher occurrence of serine in alkali stable proteins and aspertate in halophilic proteins were observed. We found that a significant number of amino acids with deviated peptide torsion observed in all the stress-tolerant proteins. We standardized the parameter with range ω < 170 in case of Trans and ω > 20 in the case of Cis^45^. The high resolution cut off value 1–1.2 Å was first attempted and noticed a halophilic protein named as High Potential Iron Sulphur represented total 9 non planar peptide bonds but only 2 was observed in case of non-halostable protein. Similarly, alkaline phosphor-serine amino-transferase (1W23) showed (Fig. 3) seven and non alkaline protein (2FYF) showed only three non planar peptide bonds. Another acidic protein acetyl-xylan esterase (1BS9) exhibited sixteen non planar peptide bonds whereas non acidic protein (1QOZ) didn’t showed non planarity.

## Discussion and Conclusion

The molecular conformational modifications against stress drove the evolutionary processes. But the information on the evolution in relation to macromolecular structural modifications is inadequate. In this background, the molecular constrains of extremophiles and non extremophiles together may provide a plausible explanation of protein structural stability. Structural plasticity of protein results molecular stability against stress which is induced by natural selection pressure. Amino acid composition described maximum uses of non polar amino acids such as glycine, alanine, valine in case of extremophiles (Fig. 1). The result from Fig. S2 and student’s t-test (P < 0.05) suggested the higher occurrence of glycine (p < 0.144), alanine (p < 0.016), valine (p < 0.048) and proline (p < 0.007) and little higher leucine (p < 0.006), isoleucine (p < 0.003)^46^ present in all the extremophilic proteins. Number of glutamine are rich in halophilic protein although it is little higher in acidic and alkaline stable proteins. This hydroxyl group containing glutamine is located predominantly at the protein surface which causes the protein flexibility^47–49^. The increased ratio of negative and positive charged amino acids that are buried on the protein surface, allow the protein to participate with ions, maintaining stability and solubility. Additionally, water binding interaction is expanded due to the presence of higher charged residue like Arg, which binds specifically with dehydrated cation which maintains a pocket of dehydration around the protein surface. Greater H-bond interactions through polar tyrosine exhibit as increased energy stabilizer for polar and charged residue. Participation of the small amino acid in the hydrophobic pocket determine with less exposure to higher concentration of ions. Lower hydrophobic contact in the core zone may increase the strength of protein stability^25^. Figure S2 along with student’s t-test of p < 0.05 may correlate the higher differences exists in between in polar (p > 0.04) and non-polar (p > 0.0004) amino acids. The isoelectric point (pI) was higher in the case of acidic proteins^50^. Lower isoelectric point in thermostable proteins occurred due to the presence of higher acidic low-charged amino acids. The average charge of all the proteins were examined (Table A-J in the Supplimentary 1 File) and it was found to be less negative value at neutral pH in case of acidophile than other proteins of extremophiles (Fig. S3). To identify the relation between sequences with structures, the global alignment (PSA) and RMSD value showed that the sequence identity has less impact on structural deviation (Table U-Y in Supplimentary 1 File). These complex structural diversities exhibited conformational plasticity and inherent rigidity against stress anomaly from high sequence similarity (Fig. 2). These structural changes may be induced by some other conformational parameter like salt bridge and intermolecular bond. The external salt bridges play a major role for stabilizing proteins in various stresses. The external salt bridge showed an effect on the surface of the protein, the properties forced to increase the strength of the interaction between charged residues (Fig. S4). The formation of the salt bridges on outer surface into the compact structure makes an association with nearby water molecules to make a potential interaction between the charged residues^43^. Various forms of stress impart protein adaptability by modulating its physico-chemical properties which eventually results in its structural adjustment. Characteristically or phenotypically adapted organism is basically adapted at the level of its proteins’ structural-functional pattern. Numerous evidences suggested that the effects of pressure and thermal stress on protein folding can be fully simulated on computers by very-fast-folding model proteins and the outcomes almost mimic a wet lab experiments. It signifies that salt-bridges and the surface ionic behavior are important determinants for protein structural adaptation^51^. Miotto *et al*. presented that, molecular interactions in thermal stress-modified protein revealed that the pattern of modification was closely related to the native**-**fold, energy**-**related parameters and to the interaction**-**networks in its structure. This can characterize differentially thermostable proteins^52^. In our current study we have shown a similar pattern of molecular adaptation in different stress-driven (acid, alkali and thermally stable) proteins. The similar nature of physico-chemical behavior changes in different conditions indicates that substrate specificity, binding and catalytic kinetics are major targets for protein modification. Local structural changes are characteristically significant in some protein modification (Dehydrins) under water related stress. This fact results in recognition specific interactions with membrane by increasing intermolecular scaffolding but not by influencing proteins tertiary structure^53^.

In our current study the resulting summary of Ramachandran plots with all of the residues in the allowed region was revealed that β-sheet left handed helix were found more condensed in the extremophiles proteins. These structural adaptations take advantage of the less exposure to the environmental stress. The structural information reveals that a complex but subtle non-planarity of the peptide bond observed, which cannot be ignored (Fig. 3). Several ultra high-resolution crystal structure (Protein deglycase 1–2.8 Å, Fibroblast growth factor 1–3.0 Å, Cytochrome P450 119–3.0 Å) of extremostable proteins showed more non planer omega than non extremophiles. Most of the small amino acids have the preference for the direction of non-planarity. Few charged amino acids like Arg and Asp play a major role in regulating peptide bond flexibility. Only neutral polar threonine performs peptide bond distortion in the case of acidophilic proteins. Nonpolar small amino acids significantly contribute into their compositional core among the extremophiles where it differs from non-extremophiles. The variation in planarity depends on the effect of stress. Furthermore, we observed that the most of the nonplanar bond, deviating by over twenty degrees from planarity which may be strongly associated with the compactness of a protein structure. The highest energy occupied π molecular orbitals through one nitrogen lone pair and minute Δω non-planarity can be dynamically ideal for protein structural stability; otherwise repulsion may reduce the interaction between amino acids^54^. During the extrapolation of the study, it should be critically correlated to the phenotypic behavior of the organism in response to the stress adapted protein modifications. When the stress adaptation is explained at molecular level at least in case of prokaryotes and especially in bacterial system it may be concluded that protein-protein interactions and protein structural dynamics promote adaptation in a large and metabolically diverse clade of the bacterial kingdom. The sigma factor (σ) regulation has been shown to control transcription in response to general stress^55^. Specific transcriptional regulation may also be the factor in eukaryotic system. Redox sensitive protein modifications (like Nrf-2, HIF-α regulation at protein and gene levels) may influence proteins functional changes at global scale. The oxidative stress theory of aging indicates that aging occur due to accumulation of oxidative damage and physico-chemical structural alterations in proteins^56^. Proteins structural modifications are of great practical importance. Cells that undergo differentiation process in response to stress, have specific pattern of protein folding modulation. Proteomic changes that occur upon differentiation explores the range of possible protein-folding modulation are controlled by the surrounding environment^57^. Similar types of behavior are evident in proteins from same category. Several prominent stress factors causes aggregation of proteins with similar properties. More specifically, it can be concluded that intrinsically aggregation proneness is also a significant factor beside the stress-specific similar nature of protein adaptation behavior. Notwithstanding, argument may be extended to the evolutionary conserved nature of protein aggregation behavior^58^.

In our current study beside physic-chemical behavior it is noticed that, physiological stress may affect in regulating peptide bond planarity locally for stabilization of protein structural conformer. Minute planarity deviations cannot be considered an exception but it may contribute a sharp structural stability against various stresses. In this regard, further studies are necessary.

## Materials and Methods

The website http://www.uniprot.org/^59^ was utilized for finding the amino acid sequences and http://www.rcsb.org^60^ was also utilized for finding the PDB file, along with fasta sequences of acidic, non-acidic, alkaline, non-alkaline, halophilic, non-halophilic, thermophilic, psychrophilic and mesophilic proteins (Vmax data collected from uniprot search engine).

### Assessment of protein in the field of amino acid composition

The compositions of amino acids were calculated and standardized by percentage using MEGA^61^. The occurrences of 200 protein homologues sequences (y-axis) were categorized (Table A-J in Supplimentary 1 File) with respect to the percentage of the abundance of particular amino acids (0–20% on x-axis) and plotted as a bar-line plot/diagram^62^. The graph represented a comparative assessment of amino acid abundance between two different types of proteins.

### Assessment of the physicochemical behavior of acidic, alkaline, halophilic, thermophilic, psychrophilic and mesophilic proteins

To study the hydrophobicity, isoelectric-point, polar, non polar and net charge characters, 200 protein sequences were taken (Table A-J in Supplimentary 1 File). The hydrophobicity of the above proteins was verified by the website http://peptide2.com/ (peptide 2.0). The isoelectric points of the above proteins were verified by the website https://www.genscript.com/ssl-bin/site2 /peptide_calculation.cgi (Genscript). The polar and nonpolar properties of the above proteins were verified by the website http://www.ebi.ac.uk (Emboss Pepstats)^63^. The net charge characters of the above proteins were calculated (Fig. S3) by using the website http://pepcalc.com/ (Innovagen).

### A statistical test on sequence and structure based allignment to identify super positioning

To compare the sequence as well as structure in terms of the protein surface and interior, the coordinates of all the extremophiles with normal were retrieved from the protein databank and their RMSD value was calculated (U-Y in Supplimentary 1 Table) with the Pymol software^64^ and the sequence similarity of proteins with their homologous normal proteins were calculated by using EMBOSS Needle^65^ tool in EBI http://www.ebi.ac.uk/Tools/psa/. 3d surface of RMSD and PSA was plotted by using NCCS (Trial version) software.

### Assessment of salt-bridges and Ramachandran plot

Due to the unavailability of acidic, alkaline, halophilic, thermophilic, psychrophilic and their homologous non-acidic, non-alkaline, non-halophilic, mesophilic proteins, only 108 (.pdb) samples were used to evaluate salt bridges and Ramachandran plot. The Salt bridges angle between participating oxygen and nitrogen atoms were calculated by running the coordinates files in VMD^66^ (K-T in Supplimentary 1 File). Only single subunit from each protein was taken and analyzed by Ramachandran plot, using VADAR^44^ provides the detail information present in each structure from their coordinate resolution (Fig. S5).

### Ethical approval

This article does not contain studies with human or animal subjects performed by any of the authors that should be approved by Ethics Committee.

## Supplementary information

Supplementary Information.

Supplementary Data Set
