# Supplementary material for: Protein stability governed by its structural plasticity is inferred by physicochemical factors and salt bridges
Source: Sci Rep. 2020 Feb 4;10:1822. doi: 10.1038/s41598-020-58825-7 (PMC7000726; doi:10.1038/s41598-020-58825-7)
Supplement: Supplementary file 1 — Supplementary Information. [file 41598_2020_58825_MOESM1_ESM.pdf]

**Title: Protein stability governed by its structural plasticity is inferred by physicochemical factors and salt bridges.**

Anindya S. Panja[1*], Smarajit Maiti[2] ,Bidyut Bandopadhyay1

From the [1] Post Graduate Department of Biotechnology, Molecular informatics Laboratory, Oriental Institute of Science and Technology, Vidyasagar University, Midnapore, West Bengal, India.
[2] Post Graduate Department of Biochemistry and Biotechnology, Cell and Molecular Therapeutics Laboratory, Oriental Institute of Science and Technology, Vidyasagar University, Midnapore, West Bengal, India.

*To whom correspondence should be addressed: Anindya S. Panja: Department of Biotechnology, Molecular informatics Laboratory, Oriental Institute of Science and Technology, Vidyasagar University, Midnapore, West Bengal, India-721102;
biotech2ani@gmail.com ; Tel.(+91)89007-89003.
**Keywords:** Extremophiles, Stress, Evolution, Salt-Bridges, Protein Structure

---

## Supporting Materials

In order to compare the amino acid compositions in terms of the protein surface and interior, seventeen acidic, twenty-two alkalines, thirty halophilic, twenty-three thermophilic, eight psychrophilic and their homologous seventeen non-acidic, twenty-two non-alkaline, twenty-two non-halophilic, twenty-four mesophilic and eight mesophilic protein sequences (Table A-J in S1 File) were taken and downloaded respectively from more than 5.5 lakhs annotated protein sequences and the percentage of 20 amino acids in each protein was calculated by accessing mega (mega5.5). In order to compare the sequence as well as structure in terms of the protein surface and interior, PDB files of eight acidic, seven alkalines, eight halophilic, twenty-four thermophilic, seven psychrophilic and their homologous eight non-acidic, seven non-alkaline, eight non-halophilic, twenty-four mesophilic and seven mesophilic proteins (U-Y in S3 File) were downloaded respectively. Due to the unavailability of acidic, alkaline, halophilic, thermophilic, psychrophilic and their homologous non-acidic, non-alkaline, non-halophilic, mesophilic proteins, only 108 (.pdb) samples among more than 1.3 lakhs molecular structure were evaluated by salt bridges and Ramachandran plot.

# Figure Legends

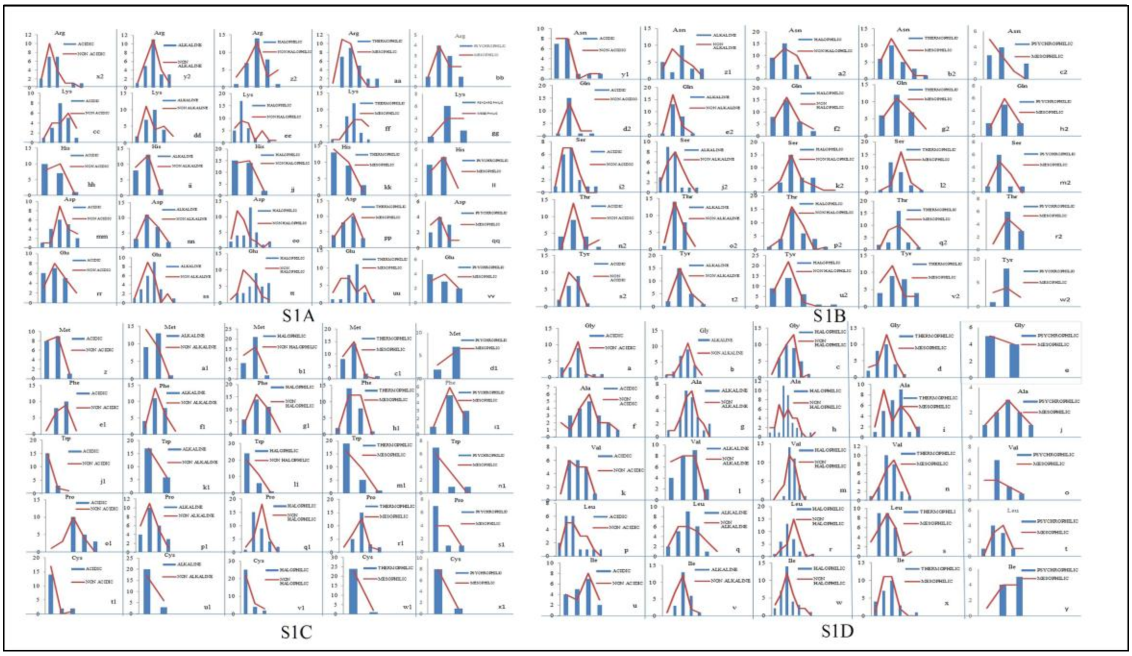


**Figure S1.** Variation of amino acid occurrence rate, representing homologous extremophile and non-extremophile sequence. The X-axis represents the ranges of amino acid residue where y axis represents the number of proteins.

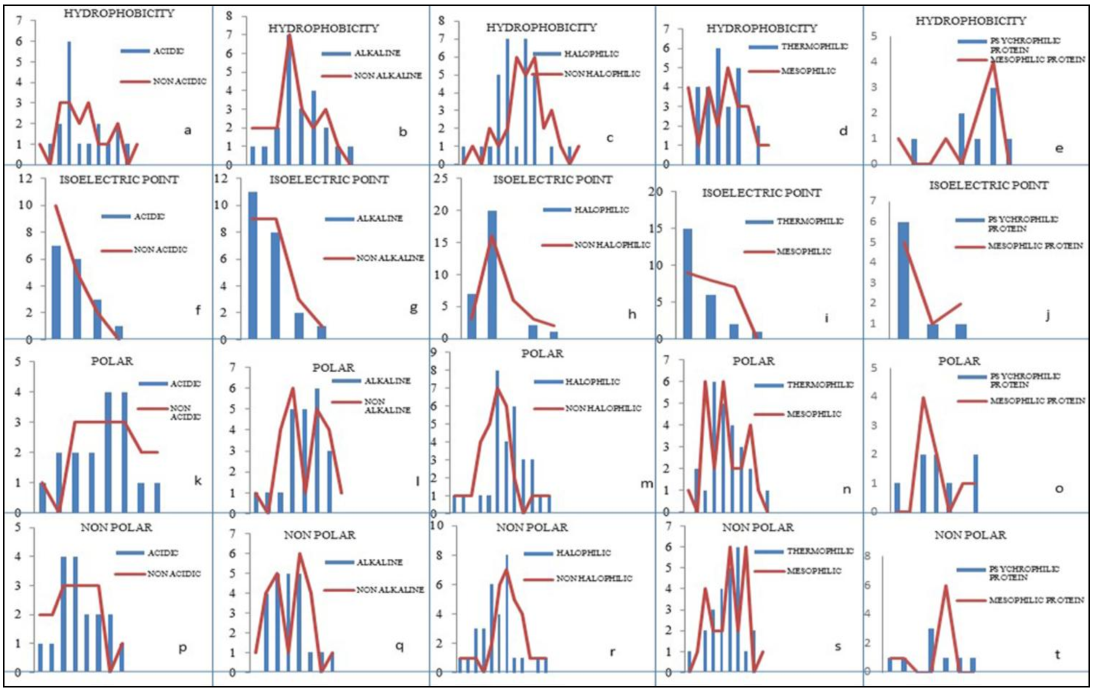


**Figure S2.** Polar, non-polar, isoelectric point and hydrophobicity is plotted against both extremophiles and non-extremophiles proteins.

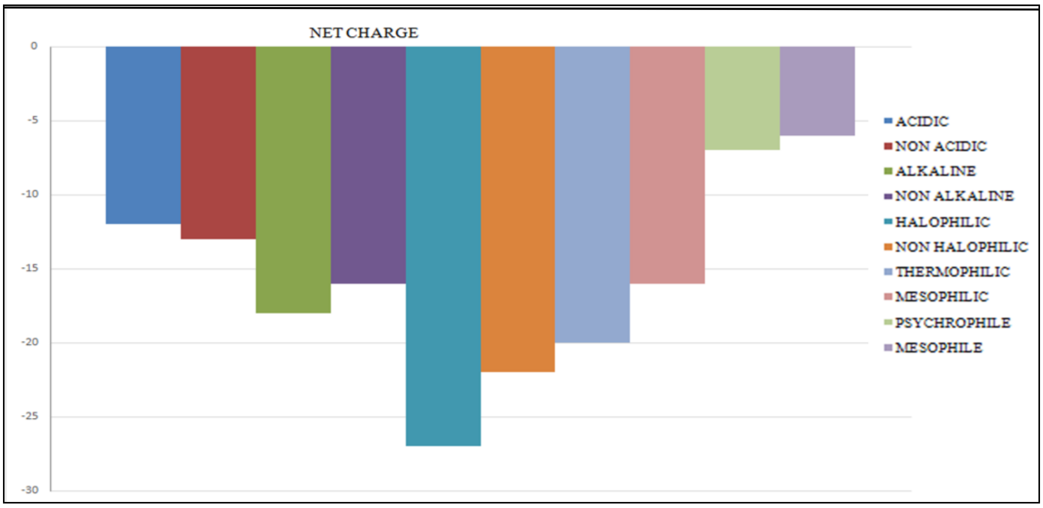


**Figure S3.** Bar diagram represents the total charge in neutral pH of the respective extremostable and normal protein.

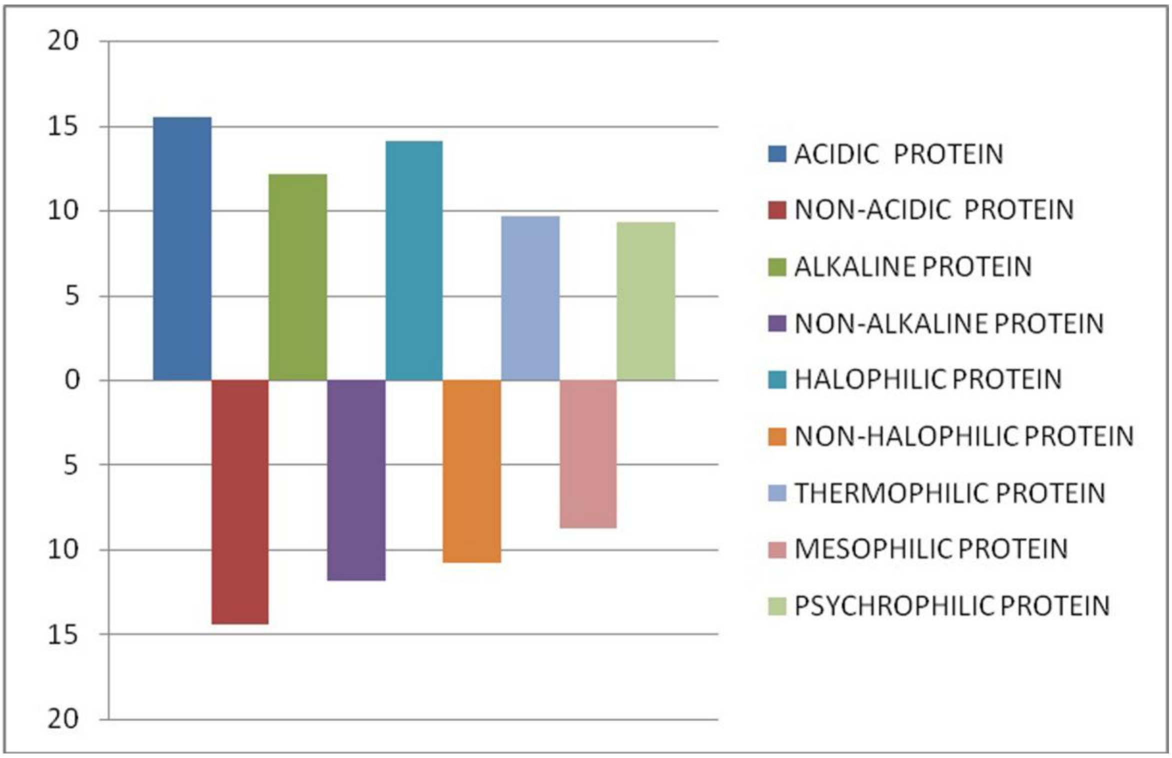


**Figure S4.** Salt bridge occurrence of the acidic, non-acidic, alkaline, non-alkaline, halophilic, non-halophilic, thermophiles and mesophiles proteins related to optimal growth.

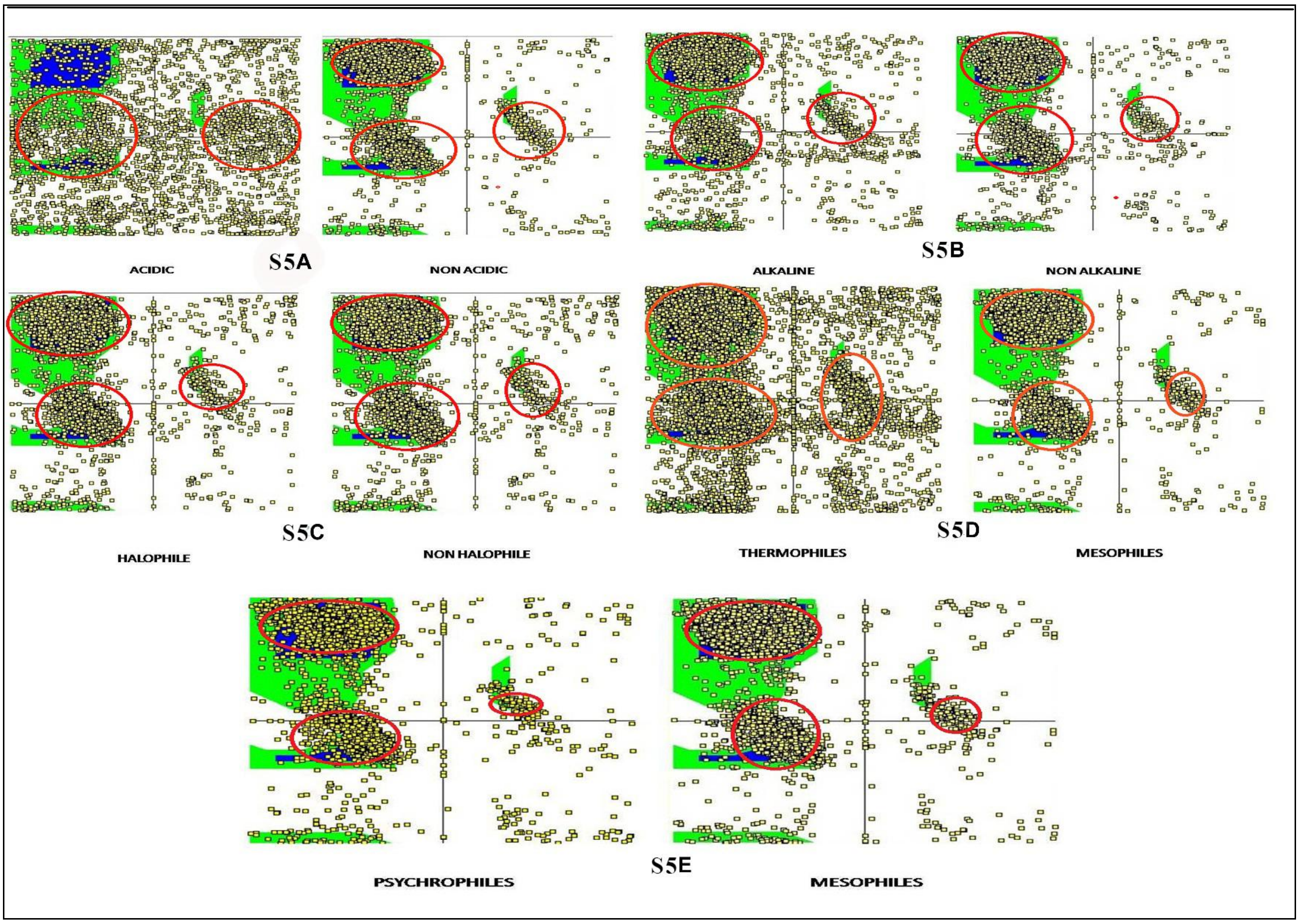


**Figure S5.** Representation of Ramachandran plot nomenclature in a different context over extremophiles and non-extremophiles proteins.

**Table S1** – Structural summary of refinement secondary structure elements based on φ and ψ angles.

| | ACIDIC | NON-ACIDIC | ALKALINE | NON-ALKALINE | HALO PHILIC | NON-HALO PHILIC | THERMO PHILIC | MESO PHILIC | PSYCHRO PHILIC |
|---|---|---|---|---|---|---|---|---|---|
| **Total no of residues checked** | 4280 | 3131 | 7278 | 6823 | 6076 | 6349 | 7328 | 7283 | 2562 |
| **% of residues in HELIX** | 33 | 23.6 | 33.6 | 30.4 | 33.4 | 32.6 | 36 | 34.4 | 42.43 |
| **% of residues in SHEET** | 26.6 | 24.6 | 19.2 | 17.4 | 27.6 | 26.6 | 28.8 | 26.8 | 29.57 |
| **Non planar peptide bonds** | 95 | 43 | 155 | 60 | 122 | 39 | 184 | 54 | 30 |
